# Reducing Pervasive False-Positive Identical-by-Descent Segments Detected by Large-Scale Pedigree Analysis

**DOI:** 10.1093/molbev/msu151

**Published:** 2014-04-30

**Authors:** Eric Y. Durand, Nicholas Eriksson, Cory Y. McLean

**Affiliations:** ^1^23andMe, Inc., Mountain View, CA

**Keywords:** identity by descent, haplotypes, population genetics, computational tools

## Abstract

Analysis of genomic segments shared identical-by-descent (IBD) between individuals is fundamental to many genetic applications, from demographic inference to estimating the heritability of diseases, but IBD detection accuracy in nonsimulated data is largely unknown. In principle, it can be evaluated using known pedigrees, as IBD segments are by definition inherited without recombination down a family tree. We extracted 25,432 genotyped European individuals containing 2,952 father–mother–child trios from the 23andMe, Inc. data set. We then used GERMLINE, a widely used IBD detection method, to detect IBD segments within this cohort. Exploiting known familial relationships, we identified a false-positive rate over 67% for 2–4 centiMorgan (cM) segments, in sharp contrast with accuracies reported in simulated data at these sizes. Nearly all false positives arose from the allowance of haplotype switch errors when detecting IBD, a necessity for retrieving long (>6 cM) segments in the presence of imperfect phasing. We introduce HaploScore, a novel, computationally efficient metric that scores IBD segments proportional to the number of switch errors they contain. Applying HaploScore filtering to the IBD data at a precision of 0.8 produced a 13-fold increase in recall when compared with length-based filtering. We replicate the false IBD findings and demonstrate the generalizability of HaploScore to alternative data sources using an independent cohort of 555 European individuals from the 1000 Genomes project. HaploScore can improve the accuracy of segments reported by any IBD detection method, provided that estimates of the genotyping error rate and switch error rate are available.

## New Approaches

Existing identical-by-descent (IBD) detection methods have largely been benchmarked using simulated data. In this study, we use data from 2,952 father–mother–child trios to analyze IBD detection method accuracy on nonsimulated data. We discover a surprisingly high rate of false positives in short segments identified by GERMLINE, a popular IBD detection method that scales well to large data sets. The false positives arise due to an algorithmic heuristic that ignores haplotype phase information. This heuristic is necessary to detect long IBD segments in the presence of switch errors. To overcome this limitation, we introduce HaploScore, a metric that quantifies the likelihood that a reported IBD segment actually matches on individual haplotypes. HaploScore effectively discriminates between true- and false-reported IBD segments and is robust to substantial parameter variation. HaploScore can be applied to IBD segments detected by any method to improve accuracy, and a Python implementation is freely available (https://github.com/23andMe/ibd, last accessed May 8, 2014).

## Introduction

IBD segments are regions of DNA between two individuals that were inherited from a recent shared common ancestor. IBD segments can be detected on high-density genetic data such as that produced by genome-wide genotyping arrays or whole-genome sequencing.

Detecting the presence and distribution of IBD segments between individuals is fundamental to many genetic applications (Browning SR and Browning BL 2012). Long-range phasing (Kong et al. 2008) uses IBD segments to resolve haplotype phasing inaccuracies. IBD segments have been used to identify disease genes (Krawitz et al. 2010; Gusev et al. 2011; Jonsson et al. 2012) and estimate the heritability of traits and common diseases (Visscher et al. 2006; Zuk et al. 2012). The lengths and distribution of IBD segments within and across populations have been used to infer demographic history (Gusev et al. 2012; Palamara et al. 2012; Ralph and Coop 2013) and identify regions under natural selection (Albrechtsen et al. 2009; Han and Abney 2013).

All methods for IBD detection ultimately try to detect a similarity between haplotypes that is statistically unlikely to occur in the absence of IBD sharing. Hidden Markov models have been used extensively for probabilistic IBD segment detection (Purcell et al. 2007; Albrechtsen et al. 2009; Browning SR and Browning BL 2010; Han and Abney 2011; Palin et al. 2011; Brown et al. 2012; Han and Abney 2013). However, these methods scale quadratically with input sample sizes and are thus not suitable for IBD detection in population-scale data sets (reviewed in Browning SR and Browning BL 2012). Nonprobabilistic IBD detection methods use a “hash-and-extend” methodology that is conceptually similar to BLAST (Altschul et al. 1990): Identical or nearly identical short haplotype match “seeds” are detected efficiently, and the seeds are extended to adjacent sites subject to heuristic constraints. These nonprobabilistic methods have the advantage that they are able to scale to much larger data sets than probabilistic methods. Implementations include GERMLINE (Gusev et al. 2009, 2012), fastIBD (Browning BL and Browning SR 2011) and RefinedIBD (Browning BL and Browning SR 2013). GERMLINE and RefinedIBD use short windows of sites as seeds, whereas fastIBD uses small segments of the inferred haplotype graph as seeds.

These three methods differ in the way that detected candidate segments are chosen to be kept as true IBD segments: FastIBD uses haplotype frequency, RefinedIBD uses a combination of segment genetic length and a likelihood ratio test, and GERMLINE uses segment length. The probabilistic refinement methods of fastIBD and RefinedIBD require a haplotype graph to be generated. Consequently, both fastIBD and RefinedIBD perform haplotype phasing in addition to IBD detection. Haplotype phasing has superlinear computational complexity (Williams et al. 2012). Current computer memory capacity constraints limit the number of individuals who can be phased together to tens of thousands of individuals. This makes the detection of all pairwise IBD segments in a cohort of over 100,000 individuals computationally infeasible using these methods because computing all pairwise IBD requires splitting the cohort into multiple smaller batches, all of which must be compared with each other, each time being phased anew. Because GERMLINE uses segment length to refine IBD segments, it does not perform genotype phasing. Consequently, detection of all pairwise IBD segments can be performed on large cohorts by phasing each individual once and then using GERMLINE to detect IBD.

IBD detection accuracy is typically assessed on simulated data, as true IBD segments can then be known precisely (Browning BL and Browning SR 2007, 2013; Albrechtsen et al. 2009; Gusev et al. 2009). However, accurate simulation of population demography is difficult (Browning SR and Browning BL 2012), and simulation parameters directly affect the estimated precision and recall of IBD detection algorithms. With a large number of father–mother–child trios, IBD detection accuracy can be estimated on nonsimulated data by examining concordance between reported IBD segments in the child and his or her parents.

In this work, we analyze the accuracy of IBD segments reported by GERMLINE because its decoupling of phasing and IBD detection make it feasible for IBD detection on population-scale data sets. We use a large cohort of trios to assess IBD segment accuracy on nonsimulated data. We perform a detailed examination of discrepant segments and present a method that substantially improves accuracy, while remaining computationally tractable for population-scale data sets. Finally, we replicate the findings using an independent cohort of individuals from the 1000 Genomes project.

## Results

### Nonsimulated Data Show Substantial Inaccuracy in Short Reported IBD Segments

To analyze IBD detection accuracy on nonsimulated data, we examined IBD segments detected in a cohort of 25,432 individuals of European ancestry that includes 2,952 distinct father–mother–child trios (the “23andMe cohort,” see Materials and Methods). By focusing specifically on segments reported between a trio child and an individual who is not a parent of that child (henceforth called “child-other” segments), IBD accuracy can be quantified: By the definition of IBD, if a child-other segment is true, at least one of the child’s parents must also share a segment IBD with the individual (henceforth called “parent-other” segments) that encompasses the child-other segment.

**Fig. 1.**
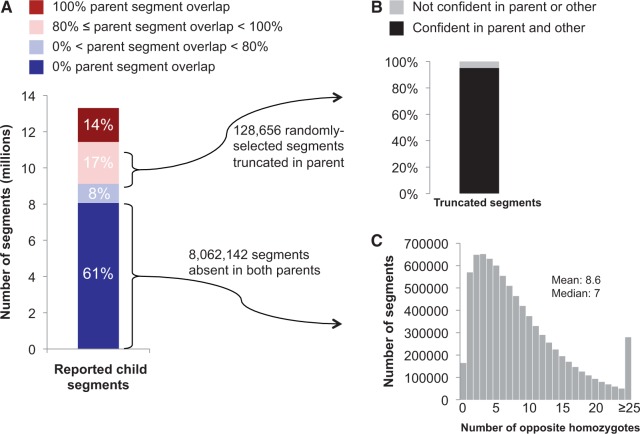
Analysis of child-other segments in parents. (*A*) The majority of child-other segments are not detected in either parent. Parent segment overlap is calculated as the percentage of sites in the child-other segment that are included in the parent-other segment. (*B*) Truncation points for parent-other segments are nearly always confidently genotyped opposite homozygote sites, consistent with false-positive IBD in the child. The opposite homozygote site causing truncation of the parent-other segment was examined in a randomly selected subset of all 3,371,616 segments with partial parent overlap. (*C*) Child-other segments with no corresponding parent-other segments contain many parent-other opposite homozygotes in the region, also consistent with false-positive IBD in the child. For each of these child-other segments, the number of opposite homozygote sites present between the parent and the other individual at that segment location is calculated separately for each parent, and the smaller is chosen as the number of opposite homozygotes in the region.

GERMLINE reported a total of 18,125,797 child-other segments in the 23andMe cohort on chromosome 21. After filtering artifactual IBD segments reported in regions of low site density, 13,307,562 child-other segments were retained for analysis. Only 14% of these child-other segments were encompassed by a parent-other segment (fig. 1*A*, supplementary fig. S1*A*, Supplementary Material online). Another 25% of child-other segments have a partial parent-other segment in which at least one segment end is truncated (fig. 1*A*, supplementary fig. S1*B*, Supplementary Material online). Segment ends imply the presence of opposite homozygote genotypes between the individuals. Opposite homozygote sites that terminate a parent-other segment exclude the possibility of child-other IBD at those sites. To determine whether truncated segment ends represented false child-other IBD or genotyping error in parent-other regions, Illumina GenCall scores were examined at the opposite homozygote sites truncating 128,656 randomly selected partial parent-other segments. Considering GenCall scores of ≥0.7 as confident genotype calls (Fan et al. 2003), over 95% of opposite homozygote sites analyzed (122,364/128,656) have confident genotype calls in both the parent and other individual. This result indicates that the majority of disagreements between child-other and parent-other segments represent false-positive IBD in the child rather than false-negative IBD in the parent (fig. 1*B*).

The remaining 61% of child-other segments have no corresponding parent-other segment (fig. 1*A*, supplementary fig. S1*C*, Supplementary Material online). All segments in this subset were analyzed to determine whether they represented false-positive child-other segments or false-negative parent-other segments by examining the number of parent-other opposite homozygote sites in the region. Nearly 98% of these child-other segments have at least one opposite homozygote site in the parent (fig. 1*C*). Given a 95% accuracy rate for parent-other opposite homozygote sites (fig. 1*B*), the probability that a region containing *N* opposite homozygote sites is actually a false-negative parent-other IBD segment was calculated as 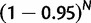. The expected fraction of false-negative parent-other segments in this subset is 0.0242, and thus, the fraction of false-positive child-other segments in this subset is 0.9758. This likely represents a conservative (i.e., low) estimate of false-positive child-other segments for two reasons: The actual genotyping accuracy is much higher than the stringent confident genotype call threshold indicates, and segments with no opposite homozygote sites can still be not shared IBD.

**Fig. 2.**
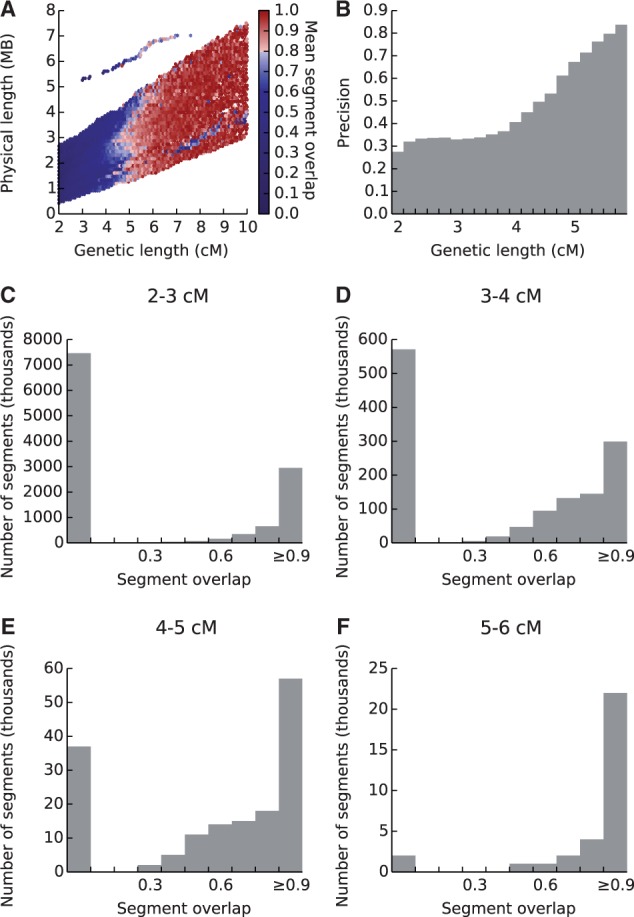
Accuracy of child-other IBD segments reported by GERMLINE. (*A*) Heat map of the mean fraction of reported child-other IBD segments contained in a corresponding parent-other segment, binned by two measures of segment length. For each child-other segment, the fraction of the segment also reported as an IBD segment between the parent and the other individual is calculated. Shown in each bin is the mean of the segment fractions calculated for all segments in the bin. (*B*) The fraction of child-other segments that are true IBD as a function of segment length. True IBD segments are defined as having at least 80% of their sites encompassed by a parent-other segment. (*C–F*) Histograms of child-other segment counts binned by segment overlap for segments of 2–3 cM (*C*), 3–4 cM (*D*), 4–5 cM (*E*), and 5–6 cM (*F*). Note the scale changes on the *y* axes: Though the fraction of true segments of length less than 3 cM is smallest, this range contains roughly 10-fold more true segments than all other length ranges combined.

The unexpectedly small number of child-other segments that are fully spanned by a corresponding parent-other segment motivated an analysis of the relationship between segment length and segment overlap. Segment overlap between parent and child was calculated based on the fraction of sites in the child-other segment (supplementary fig. S1, Supplementary Material online). Segments were segregated by genetic and physical lengths, and the average segment overlap of all segments in each bin was calculated (fig. 2*A*). Genetic length is a more reliable indicator of average segment overlap than physical length, and segments longer than 6 cM generally show a high degree of overlap. However, the average overlap drops rapidly as segment length is reduced (fig. 2*A*).

IBD accuracy was estimated by considering child-other segments with substantial parent-other segment overlap as true IBD. Because precise determination of IBD endpoints from genotype data is difficult (Browning BL and Browning SR 2013), a threshold of 80% segment overlap was used to classify a segment as true IBD. Using this criterion, more than 67% of all reported segments shorter than 4 cM are false-positive child segments (fig. 2*B*). Figure 2*C–F* show the IBD segment overlap distributions segregated by genetic length. Most 2–3 cM segments are erroneous (fig. 2*C*), and only segments longer than 5 cM have a negligible number of false positives (fig. 2*F*). Indeed, when filtering solely by genetic length, all segments shorter than 5 cM must be discarded to achieve a precision value of 0.8 (supplementary fig. S2, Supplementary Material online). However, because there are many more short segments (supplementary fig. S3, Supplementary Material online), eliminating all segments shorter than 5 cM eliminates 99% of all true IBD segments, a dramatic loss in recall (supplementary fig. S2, Supplementary Material online). In the next section, we investigate the properties of true IBD segments of all lengths and contrast them with erroneous segments.

### Overly Permissive Diplotype Matching Causes Reported Segment Inaccuracy

IBD segments are shared between two individual haplotypes. Thus, if the phase of each individual genotype was known, IBD detection algorithms could in principle analyze each individual haplotype independently. However, for individuals without a genotyped pedigree, genotypes have to be phased statistically, where switch errors occur at an appreciable frequency (supplementary fig. S4, Supplementary Material online). Examination of only haplotypes in the presence of switch errors is known to reduce power to detect IBD, especially for long segments (Browning BL and Browning SR 2013), because they are likely to harbor more switch errors than short segments. Thus, GERMLINE (and many other IBD detection methods) matches IBD segments between individual diplotypes, trying to allow for a moderate number of switches between individuals’ haplotypes. In practice, this is achieved by allowing haplotype match seeds to extend until an opposite homozygous site is met. There are two potential issues with this approach that could lead to inconsistent segment reporting between parent and child and are explored further below.

Detection of child-other segments with a truncated or absent corresponding parent-other segment could arise from the haplotype matching between the child and the other individual, but a switch error in the parent causing the corresponding haplotype to not match between the parent and the other individual. To investigate this potential error source, all 2,952 trios were trio-phased using the laws of Mendelian inheritance, and then IBD detection was performed as before. Trio-phasing ensures that children and parents are phased essentially perfectly (i.e., up to recombination events), eliminating haplotype discrepancies between parent and child as a source of segment discrepancies. The number and accuracy of child-other segments using trio-phased data is nearly identical to that of BEAGLE-phased data, showing that parent–child haplotype discrepancies contribute a negligible amount toward discrepant segments (supplementary fig. S5, Supplementary Material online).

Alternatively, child-other segments with no corresponding parent-other segment could be false reported IBD between the child and the other individual due to overly permissive diplotype matching. To examine this possibility, each full 100-site window in all 13,307,562 child-other segments was analyzed (63,542,380 total windows) to see whether the window satisfied the diplotype match criterion and the haplotype match criterion between the child and the other individual and between the parent and the other individual. The analysis was segregated by windows contained within corresponding parent-other segments (likely true IBD) and windows that are not contained within corresponding parent-other segments (false IBD). The diplotype match criterion is satisfied in the child in 97.6% of windows contained within parent-other segments (table 1) and in 97.5% of windows not contained within parent-other segments (table 2). Approximately 67.7% of windows contained within both child-other and parent-other segments satisfy the haplotype match criterion for IBD in the child (table 1), consistent with true IBD given the window size and empirical switch error rate (supplementary fig. S4, Supplementary Material online). In contrast, only 44.2% of windows not contained within a parent-other segment satisfy the haplotype match criterion for IBD in the child (table 2), a substantial reduction (binomial 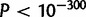).

**Table 1.** Haplotype and Diplotype Window Matches in Child-Other Segments Contained within a Corresponding Parent-Other Segment.

	Child Diplo	Child Haplo	Child Both	Total
Par none	0	0	0	0
Par diplo	6,283,300	56,393	1,045,425	7,385,118
Par haplo	57,353	243,447	236,157	536,957
Par both	1,098,359	243,490	13,733,586	15,075,435
Total	7,439,012	543,330	15,015,168	22,997,510

Note.—Par, parent; diplo, diplotype match only; haplo, haplotype match only.

**Table 2.** Haplotype and Diplotype Window Matches in Child-Other Segments Not Contained within a Corresponding Parent-Other Segment.

	Child Diplo	Child Haplo	Child Both	Total
Par none	14,055,602	483,921	5,853,193	20,392,716
Par diplo	7,574,059	77,905	2,068,399	9,720,363
Par haplo	82,378	243,698	372,885	698,961
Par both	931,599	222,127	8,579,104	9,732,830
Total	22,643,638	1,027,651	16,873,581	40,544,870

Note.—Par, parent; diplo, diplotype match only; haplo, haplotype match only.

The poor precision in short segments is thus due to the allowance of diplotype-only matches within the IBD detection algorithm. However, allowing diplotype-only matches is necessary for detection of long segments due to imperfect haplotype phasing (Gusev et al. 2012). The substantial reduction in windows matching haplotypes in regions of false IBD suggests a haplotype-based metric that is robust to switch errors could improve precision of reported IBD without the loss of recall incurred by haplotype-only IBD detection mechanisms.

### A Haplotype-Based Metric to Identify True IBD Segments

**Fig. 3.**
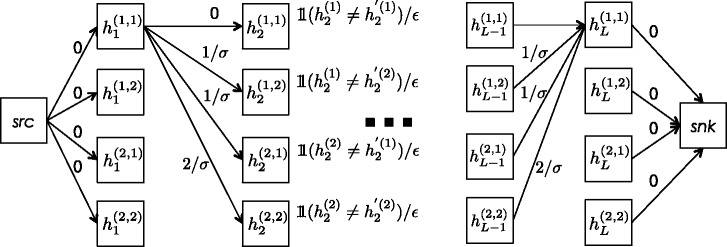
Graph illustrating the HaploScore computation. The HaploScore for an IBD segment of length *L* can be represented as the minimum-cost path through the above DAG, where *ε* denotes the probability of a genotyping error and *σ* denotes the probability of a switch error at any given site. The DAG has one source, one sink, and one level per genotyped site in the IBD segment. At each level *l*, the DAG contains four nodes, indicating the haplotype configuration at site *l*. Each node has weight 0 if the two corresponding alleles are the same, or 1/*ε* if they are different. Each node in level *l* has four outgoing directed edges, one to each of the four nodes in level *l* + 1. The edge weights are 0, 1/*σ*, or 2/*σ*, depending on whether 0, 1, or 2 switch errors are necessary to explain the transition. For clarity, some edges are omitted in this figure. The source node *src* has four outgoing directed edges with weight 0, one to each of the four nodes in level 1. Each node in level *L* has one outgoing directed edge to the sink node *snk* with weight 0.

IBD is fundamentally a property of haplotypes, not diplotypes. Consequently, true IBD should appear consistent with haplotype matches, modulo expected genotyping and switch errors. We introduce HaploScore as a measure of haplotype IBD likelihood: Given a genotyping error rate per site *ε* and a switch error rate per site *σ*, the HaploScore for a candidate IBD segment *S* is

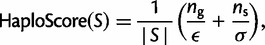

where |*S*| is the number of genotyped sites in *S,* and *n*_g_ and *n*_s_ are the number of genotyping and switch errors, respectively, that together minimize the score while reconciling the segment as matching across a single haplotype in both individuals (fig. 3). Conceptually, HaploScore is a measure of the ratio of observed and expected genotyping and switch errors. In segments falsely reported as IBD, a larger-than-expected number of genotyping and switch errors may be required to reconcile the segments as matching across individual haplotypes, and their HaploScores will be large.

**Fig. 4.**
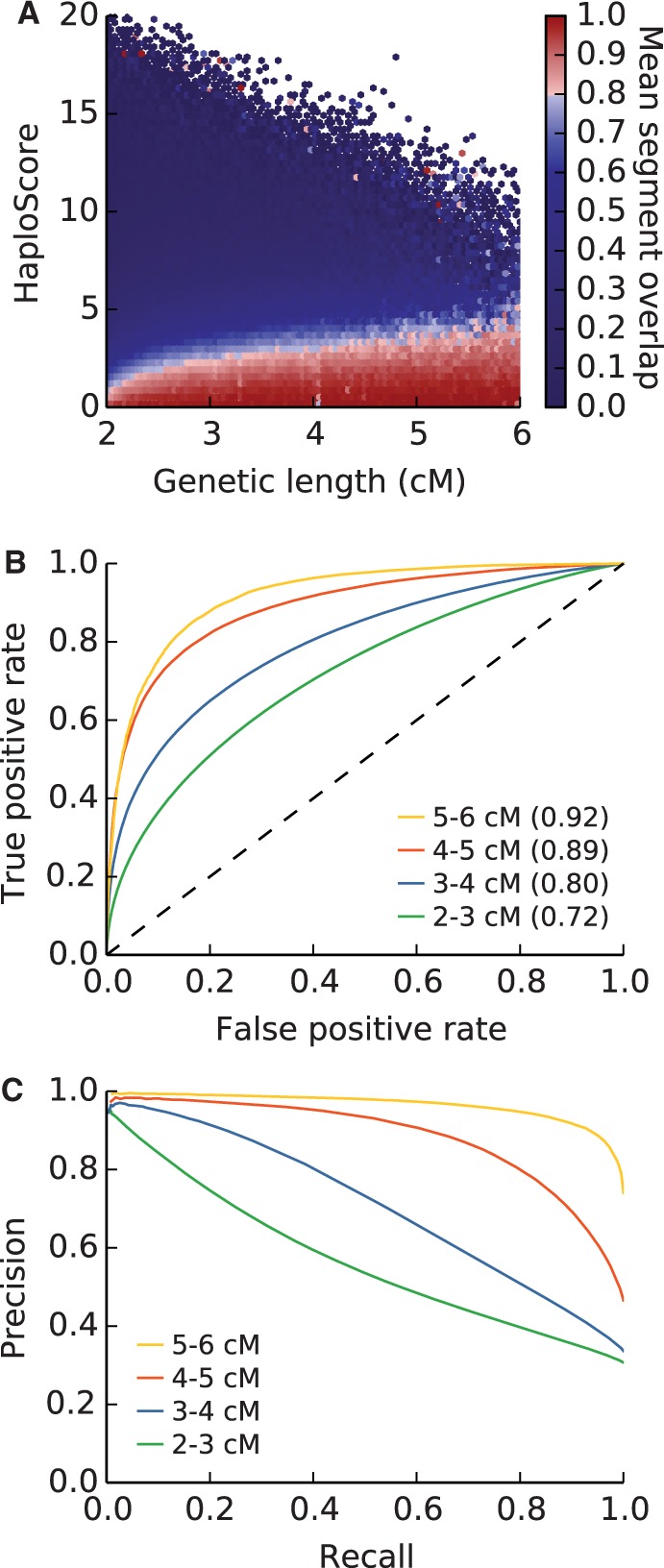
Improving detection of true IBD segments using HaploScore. (*A*) Heat map of the mean fraction of reported IBD segments found in parents, binned by segment genetic length and HaploScore. Calculations are performed as in figure 2*A*. (*B*) Receiver operating characteristic for reported IBD segments of various lengths, discriminating by HaploScore. True IBD is defined as in figure 2*B*. The dashed black line indicates the no-discrimination line. The area under each curve is parenthesized in its legend entry. (*C*) Precision-recall plot for child-other segments binned by segment length.

Genotyping and switch error rates per site were estimated from the data to be *ε* = 0.0075 and *σ* = 0.003 (Materials and Methods). Using those parameters, HaploScore was calculated on all segments shorter than 6 cM. To investigate whether HaploScore behaves differently between true and false IBD, we plotted a heat map of IBD segment overlap as a function of segment genetic length and HaploScore values. HaploScore effectively discriminates true and false IBD segments at all lengths (fig. 4*A*). Indeed, the relationship between HaploScore and mean segment overlap is nearly monotonic, drawing a clear boundary between segments with at least 80% overlap and others at all genetic lengths.

In addition, we assessed the power of HaploScore as a binary classifier to decide if an IBD segment is true. We varied a HaploScore threshold from 0 to 22 (the maximum observed HaploScore value on chromosome 21) and classified segments with a HaploScore value smaller than the threshold as true IBD. We then computed the true-positive and false-positive rates at each HaploScore threshold. HaploScore performed well as a binary classifier at all genetic lengths, achieving an area under the receiver operating characteristic curve (AUC) greater than 0.8 for segments longer than 3 cM (fig. 4*B*). At all levels of precision, power increased as segment length increased, owing at least in part to the general positive correlation between segment length and number of sites in the segment. Importantly, and in sharp contrast with length-based filtering (supplementary fig. S2, Supplementary Material online), HaploScore-based filtering retains many segments shorter than 5 cM at a precision of 0.8 (fig. 4*C*). Recall of HaploScore-based filtering at 0.8 precision is 0.19, a 13-fold increase compared with length-based filtering.

### Robustness of Results to HaploScore Parameter Variation

**Fig. 5.**
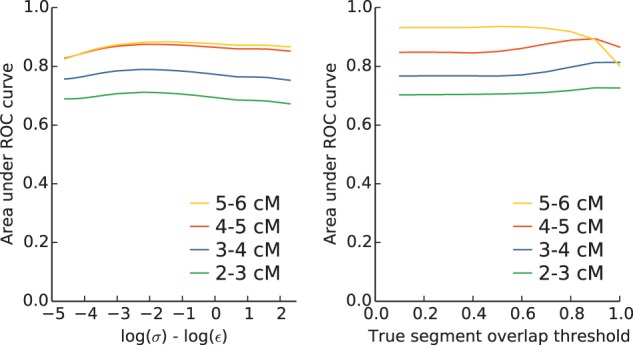
HaploScore is robust to a wide range of input parameters. (*A*) AUCs for a range of genotyping to switch error rate ratios. We varied the switch error rate *σ* relative to the genotyping error rate *ε*. For each value of *σ*, we evaluated the resulting AUC discriminating by HaploScore, where we defined true-positive segments as having a segment overlap of at least 0.80. (*B*) AUCs for a range of segment overlap values required to classify a segment as a true positive. For each of ten different segment overlap thresholds (0.1, … ,1.0), we classified true-positive segments and calculated the resulting AUC discriminating by HaploScore.

HaploScore is a function of two parameters: The genotyping error rate *ε* and the switch error rate *σ*. However, only the ratio of the two parameters affects the behavior of the score. To assess the robustness of HaploScore to varying parameters, a grid search was performed in which *ε* was fixed at 0.0075, *σ* was varied three orders of magnitude from *ε*/100 to 10*ε*, and the AUC was computed at each grid point (fig. 5*A*). As expected, performance was strongest when the ratio of the parameters was near its true value. However, the performance degradation was modest across the wide range of parameter ratio values examined, with the AUC dropping by less than 2% at worst.

### Robustness of Results to True IBD Definition

In all analyses above, true IBD segments were defined as child-other segments that have at least 80% parent-other segment overlap. To assess the robustness of HaploScore to different true IBD definitions, a grid search was performed in which the definition of true IBD was varied from 10% to 100% parent-other segment overlap in increments of 10%. The AUC was computed at each grid point (fig. 5*B*). Performance was generally stable for all segment lengths and true IBD definitions, with the exception of 5–6 cM segments at 100% overlap, where performance degraded appreciably. This is likely due at least in part to the inherent bias for longer segments to have more sites at which premature truncation of detected IBD segments can arise from genotyping or switch errors.

### Robustness of Results to Genome-Wide IBD Identification

To confirm that the results presented are not due to particular genomic features of chromosome 21, chromosome 10 was analyzed on the full cohort using the same parameters (*ε* = 0.0075, *σ* = 0.003, 80% segment overlap defined true IBD). The results were qualitatively similar to chromosome 21, showing that the HaploScore methodology is extensible genome wide (supplementary fig. S6, Supplementary Material online). In addition, IBD segments were examined on all autosomes in the subset of all individuals comprising the 2,952 unrelated trios. No substantial deviations in performance were observed (not shown).

### Filtering Spurious Reported IBD Segments Using HaploScore

HaploScore can be used to filter out spurious segments reported by an IBD detection algorithm as an efficient postprocessing step. The reduced power to detect short segments requires more stringent HaploScore threshold values for shorter segments to achieve a similar precision value as for longer segments (fig. 4). Because HaploScore provides a way to rank segments, the trade-off between precision and recall can be tuned to the needs of the particular downstream application.

**Fig. 6.**
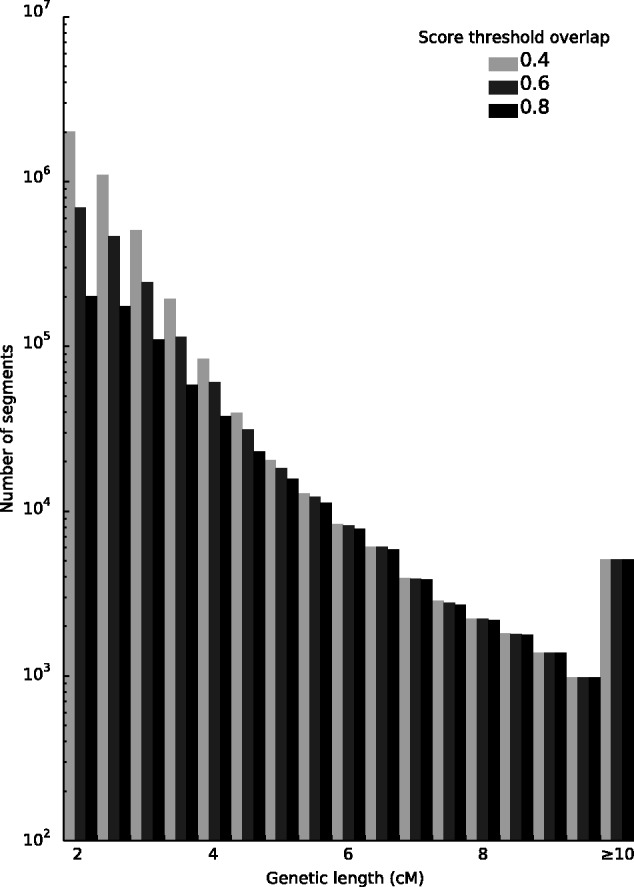
Segment detection and HaploScore filtering results. Histogram of number of segments reported after filtering at three different HaploScore thresholds, 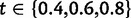. Each threshold *t* corresponds to a genetic-length-specific array of maximal HaploScores allowed to retain all segments with mean segment overlap of at least *t*, as described in the HaploScore threshold matrix generation section of Materials and Methods. Note that the *y* axis is on a log scale.

HaploScore threshold values to ensure particular average overlap values of resultant segments were generated (see Materials and Methods), and three separate filtering results are shown in figure 6. Notably, more stringent filtering parameters have the largest effect on short segments and have nearly the same effect as lenient filtering parameters for segments over 5 cM (fig. 6). This result is intuitive, as the short reported segments are enriched for false positives (fig. 2*C–F*).

### Robustness of Results to Alternate Individuals and Genotyping Platforms

To assess the robustness of the findings in an alternative population, a cohort of 555 European individuals including 52 father–mother–child trios genotyped as part of the 1000 Genomes project (1000 Genomes Project Consortium 2012) were analyzed (the “1000 Genomes cohort,” supplementary table S1, Supplementary Material online). Individuals in the 1000 Genomes cohort were genotyped on the Illumina HumanOmni2.5-Quad v1-0 B SNP array and as such provide an independent sample set from which to assess the generalizability of our results to additional individuals and alternative genotyping platforms.

GERMLINE reported a total of 6,585 child-other segments on chromosome 21 in the 1000 Genomes cohort. After filtering artifactual IBD segments reported in regions of low site density, 5,770 child-other segments were retained for analysis. The number of child-other segments detected in the 1000 Genomes cohort is much smaller than in the 23andMe cohort (5,770 versus 13,307,562 candidate segments) because the 1000 Genomes cohort is much smaller. However, the rate of candidate segment detection is similar: In the 1000 Genomes cohort, there are 5,770 segments for 52 × 552 child-other pairs, resulting in an average of 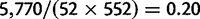 child-other segments per trio. In the 23andMe cohort, the corresponding rate is 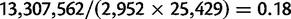 child-other segments per trio.

Analyses of child-other segments detected in the 1000 Genomes cohort were performed analogously to those in the 23andMe cohort. Only 12% of child-other segments were encompassed by a parent-other segment, 20% of child-other segments have a partial parent-other segment in which at least one segment end is truncated, and the remaining 68% of child-other segments have no corresponding parent-other segment (supplementary fig. S7*A*, Supplementary Material online). Analysis of truncated segments in the 1000 Genomes cohort also strongly suggests that false child-other IBD accounts for most discrepant segments, as 92% of opposite homozygote sites that truncate the 1,174 truncated segments have confident genotype calls in both the parent and other individual (supplementary fig. S7*B*, Supplementary Material online). Finally, in the 68% of child-other segments that have no corresponding parent-other segment, over 99% contain at least one opposite homozygote site in the parent (supplementary fig. S7*C*, Supplementary Material online). Taken together, these results show that the 1000 Genomes cohort is also rife with false-positive IBD, and despite the different genotyping platform used, the error profile in the 1000 Genomes cohort is qualitatively very similar to that in the 23andMe cohort (fig. 1).

Examination of the relationship between segment length and segment overlap in the 1000 Genomes cohort indicates similar general trends as those discovered in the 23andMe cohort (compare supplementary fig. S8, Supplementary Material online, and fig. 2), though the smaller number of segments makes the results more noisy. Comparison of all 44,542 full 100-site windows in the 5,770 child-other segments shows that overly permissive diplotype matching causes false reported IBD segments: The diplotype match criterion is satisfied in 97.1% of windows contained within parent-other segments and in 96.5% of windows not contained within parent-other segments, whereas the haplotype match criterion is satisfied in 68.7% of windows contained within parent-other segments but in only 51.1% of windows not contained within parent-other segments (supplementary table S2, Supplementary Material online), a substantial reduction (binomial 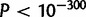).

Finally, the performance of HaploScore in segregating true- and false-reported IBD was analyzed in the 1000 Genomes cohort. The switch error rate was estimated from the data to be *σ* = 0.003, and the genotyping error rate was estimated to be *ε* = 0.0075. Similar trends are present in the 1000 Genomes cohort as are in the 23andMe cohort (compare supplementary fig. S9, Supplementary Material online, and fig. 4). The small number of child-other segments analyzed in the 1000 Genomes cohort causes somewhat noisy results, but the effectiveness of HaploScore as a discriminator between true- and false-positive IBD is readily apparent.

## Discussion

The usage of IBD segments in genetic analyses will become increasingly common as the number of individuals with their genetic composition known increases. Because of the inherently quadratic nature of IBD detection between all pairs of individuals in a cohort, nonprobabilistic methods are required to keep the computational burden as low as possible. However, effective filtering methods are required to ensure reported IBD segments are accurate.

Using the laws of Mendelian inheritance is an effective way to avoid modeling complex demographic history when evaluating the accuracy of population genetics methods including IBD detection and local ancestry inference (Pasaniuc et al. 2013). By using known familial relationships in a large set of trios, we were able to analyze the accuracy of IBD segments reported by GERMLINE on nonsimulated data. We found a surprisingly large number of false-positive short segments and showed that these false positives arose due to the diplotype-based IBD detection mechanism introduced to detect long IBD segments in the presence of phasing switch errors (Gusev et al. 2012). We introduced a haplotype-based metric, HaploScore, that effectively discriminates between true- and false-reported IBD segments. We also investigated a likelihood-ratio-based metric but found it less effective than HaploScore (supplementary text, Supplementary Material online).

Importantly, HaploScore can be computed efficiently using dynamic programming (in O(|*S*|) time per segment, see Materials and Methods). This suggests a strategy for accurate IBD detection in population-scale data sets: Detect candidate segments using a nonprobabilistic IBD detection method with relatively permissive parameters and then cull true segments using HaploScore filtering. In addition, HaploScore can be applied as a postprocessing step to existing genotyping- and sequencing-based IBD segments, provided that an estimate of the switch error rate and the genotyping error rate are available.

Achieving optimal HaploScore performance in a different population cohort or when using an alternative genotyping platform depends on being able to accurately estimate the genotyping and switch error rates of the data. Genotyping error rates can be estimated in any cohort by methods such as repeat genotyping (Pompanon et al. 2005). Although accurate determination of switch error rates currently requires trios or orthogonal analysis methods such as phased sequencing (Voskoboynik et al. 2013), the robustness of HaploScore to substantial variations in the parameter ratio indicates that it should be extensible to non-European populations, genotyping platforms of different marker density, or even sequencing-based assays. Indeed, we demonstrated the robustness and generalizability of HaploScore by analyzing an independent cohort of 555 European individuals from the 1000 Genomes project who were genotyped on a chip nearly twice as dense as the 23andMe chip. Although the smaller sample size of the 1000 Genomes cohort produced noisier results, all major findings of the analysis of the 23andMe cohort were replicated in the 1000 Genomes cohort.

Python code implementing HaploScore filtering and the IBD segments analyzed herein are freely available (https://github.com/23andMe/ibd, last accessed May 8, 2014).

## Materials and Methods

### Cohort Description

The 23andMe cohort analyzed comprises 25,432 customers of 23andMe, Inc., a personal genetics company, who were genotyped on the Illumina HumanOmniExpress + BeadChip as part of the 23andMe Personal Genome Service. The chip contains approximately 1,000,000 sites genome wide (Hinds et al. 2013). Individuals were selected for having more than 97% European ancestry as described previously (Hinds et al. 2013). The 23andMe cohort includes 2,952 distinct father–mother–child trios identified by IBD sharing (Henn et al. 2012). Parent–child relationships were defined as having at least 85% of the genetic length of the genome shared on at least one haplotype and no more than 10% of the genetic length of the genome shared on both haplotypes. Parent–parent relationships were defined as having at most 20% of the genetic length of the genome shared on at least one haplotype.

The 1000 Genomes cohort analyzed comprises 555 individuals from five European populations who were genotyped on the Illumina HumanOmni2.5-Quad v1-0 B SNP array as described previously (1000 Genomes Project Consortium 2012) (samples available at ftp://ftp-trace.ncbi.nih.gov/ 1000genomes/ftp/technical/working/20120131_omni_genotypes_and_intensities/Omni25_genotypes_2141_samples.b37.vcf.gz, last accessed May 8, 2014). The 1000 Genomes cohort includes 52 distinct father–mother–child trios identified within the 1000 Genomes project (metadata available at ftp://ftp-trace.ncbi.nih.gov/1000genomes/ftp/technical/working/20130606_sample_info/20130606_sample_info.txt, last accessed May 8, 2014) and which we validated independently by IBD sharing (supplementary table S1, Supplementary Material online). All members of the 1000 Genomes cohort were verified to not be present in the 23andMe cohort.

### Ethics Statement

All participants in the 23andMe cohort were drawn from the customer base of 23andMe, Inc., a consumer genetics company. Informed consent was obtained. Individual-level genotype data are protected pursuant to 23andMe’s research protocol approved by the external AAHRPP-accredited IRB, Ethical & Independent Review Services (E&I Review), and cannot be released. Details of the 1000 Genomes cohort are described elsewhere (1000 Genomes Project Consortium 2012).

### IBD Detection

#### The 23andMe Cohort

Genotypes of all individuals included in the 23andMe cohort were phased using BEAGLE (Browning SR and Browning BL 2007) version 3.3.1 in batches of 8,000–9,000 individuals as described previously (Hinds et al. 2013). In each batch, we excluded sites with minor allele frequency less than 0.001, Hardy–Weinberg equilibrium 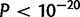, call rate < 95%, or large allele frequency discrepancies compared with the 1000 Genomes Project reference data. Input haplotypes were restricted to sites present in the intersection of all batch-filtered sites and resulted in 12,881 sites on chromosome 21 and 48,372 sites on chromosome 10.

For each of the 2,952 trio children, candidate IBD segments were calculated between the child and all 25,429 other individuals who were not the parents of that child. For each of the 5,904 (= 2 × 2,952) trio parents, candidate IBD segments were calculated between the parent and all 25,430 other individuals who were not the child of that parent. All candidate IBD segments were calculated using the GERMLINE (Gusev et al. 2009) algorithm with the parameters -bits 100 -err_hom 2 -err_het 0 -w_extend -min_m 2 -map <geneticmap>, corresponding to the empirical genotyping and switch error rates of the data (see HaploScore parameter estimation). The genetic map used was generated by the Phase II HapMap (Frazer et al. 2007) (available at http://hapmap.ncbi.nlm.nih.gov/downloads/recombination/2011-01_phaseII_B37/genetic_map_HapMapII_GRCh37.tar.gz, last accessed May 7, 2014) and lifted over to NCBI Build GRCh37 coordinates using the UCSC Genome Browser (Kent et al. 2002) liftOver tool. To omit clearly artifactual candidate IBD segments arising from sequence assembly gaps and platform effects, candidate segments were filtered by site density (Zhuang et al. 2012). Segments with a site density (measured in sites/cM) in the lowest 10% of all 1 cM windows on the chromosome were omitted. All remaining candidate IBD segments were retained.

#### The 1000 Genomes Cohort

Genotypes of all 555 individuals in the 1000 Genomes cohort were phased using BEAGLE (Browning SR and Browning BL 2007) version 3.3.1 in a single batch. Windows of 3,000 sites that overlapped by 100 sites were stitched together as described previously (Hinds et al. 2013). Sites that were not polymorphic in the 555 individuals examined had a 1000-Genomes-reported Hardy–Weinberg equilibrium 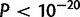 or a call rate within the 555 individuals examined less than 95% were excluded, resulting in 23,142 sites on chromosome 21. GenCall genotype scores were set to 0 for all sites not called in each individual.

Candidate IBD segments were identified and filtered identically to those found in the 23andMe cohort described above.

### HaploScore Description and Computational Complexity

HaploScore provides a metric by which to rank the likelihood that a stretch of DNA is inherited IBD between two individuals or not. Let *ε* and *σ* denote the probability of a genotyping error and a switch error at any given site, respectively. The HaploScore for a candidate IBD segment *S* is
(1)
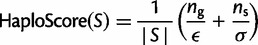

where |*S*| is the number of genotyped sites in *S,* and *n*_g_ and *n*_s_ are the number of genotyping and switch errors, respectively, that together minimize the score while reconciling the segment as matching across a single haplotype in both individuals.

Finding the HaploScore (i.e., the optimal values of *n*_g_ and *n*_s_ subject to the constraints) can be viewed as finding the minimum-cost path through the directed acyclic graph (DAG) described below (fig. 3).

Let *G* be a DAG with a single source node and a single sink node. Between the source and the sink, the graph has |*S*| levels, one per genotyped site in segment *S*. Each of these |*S*| levels has four nodes, one for each possible haplotype configuration. Each node in level *l* has four outgoing directed edges, one to each node in level *l* + 1. Below, we use the same notation for nodes and their weights.

At any level *l*, let 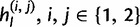 denote the four possible haplotype configurations of an IBD match. The nodes are weighted as follows:
(2)
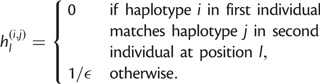



Let 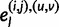 denote the weight of the edge between nodes 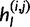 and 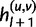. Edges are weighted as follows:
(3)
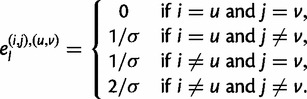



The weights of the four edges from the source node to the nodes in the first level, as well as the weights from the nodes in level |*S*| to the sink node, are set to 0. The cost of a path in *G* is defined as the sum of the weights of the edges and nodes it traverses.

HaploScore(*S*) is equal to the smallest of all path costs from the source to the sink. It can be efficiently computed using dynamic programming by noting that the smallest cost from the source to level *l* + 1 in the graph can easily be inferred from the smallest cost from the source to level *l*. Let 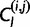 denote the smallest cost from the source to haplotype configuration (*i*, *j*) at level *l*. Then,
(4)
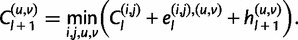



The minimum cost to reach level *l*, 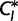, is then
(5)
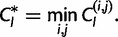



The above equations clearly show that computing HaploScore(*S*) involves 16 comparisons at each genotyped site in *S*. Thus, the complexity of computing HaploScore(*S*) is at most 16|*S*|. Performance can be further improved when filtering by HaploScore by terminating computation as soon as a segment’s HaploScore becomes too high to satisfy the maximum value threshold.

### HaploScore Parameter Estimation

HaploScore uses two parameters, the genotyping error rate per site *ε* and the switch error rate per site *σ*. Analyses of genotyping chip accuracy (Paynter et al. 2006) and internal comparisons between genotype and whole-genome sequencing data verify that the genotyping error rate is less than 1% (not shown). To estimate the empirical switch error rate per site, all 2,952 trios in the 23andMe cohort were trio-phased using the laws of Mendelian inheritance, and the results for all children were compared with their BEAGLE-phased haplotypes, assuming that the trio-phased haplotypes represented the true phase. The average per-site switch error rate ranged from 0.0019 (on chromosome 6) to 0.0043 (on chromosome 19) but deviated only modestly from a constant rate on each chromosome (supplementary fig. S4, Supplementary Material online).

The switch error rate calculation process described above was performed independently on the 1000 Genomes cohort. A total of 3,629 switch errors were detected in the 52 trio children over 23,142 sites. This corresponds to an individual switch error rate per site of 3,629/(52 × 23,142) = 0.003.

### HaploScore Threshold Matrix Generation

A matrix of HaploScore thresholds was generated in the following manner: All segments were binned by genetic length in 0.1 cM increments from 2 to 10 cM. In each length bin, segments were segregated by their segment overlap into 100 equally sized overlap bins. The score threshold in each overlap bin was initially set to be the average HaploScore of all segments within the bin. To ensure monotonicity, the score threshold was then taken to be the maximum of the scores in all bins of equal or higher overlap at that segment length. A file containing the maximum HaploScore value thresholds calculated for all genetic lengths and mean overlap values in the 23andMe cohort is available (supplementary file S1, Supplementary Material online).

## Supplementary Material

Supplementary figures S1–S10, text, file S1, and tables S1–S4 are available at *Molecular Biology and Evolution* online (http://www.mbe.oxfordjournals.org/).

Supplementary Data
