# Supplementary material for: Reducing Pervasive False-Positive Identical-by-Descent Segments Detected by Large-Scale Pedigree Analysis
Source: Mol Biol Evol. 2014 Apr 30;31(8):2212–22. doi: 10.1093/molbev/msu151 (PMC4104314; doi:10.1093/molbev/msu151)
Supplement: Supplementary Data [file supp_31_8_2212__index.html]

Reducing pervasive false positive identical-by-descent segments detected by large-scale pedigree analysis — Reducing Pervasive False-Positive Identical-by-Descent Segments Detected by Large-Scale Pedigree Analysis — Reducing Pervasive False-Positive Identical-by-Descent Segments Detected by Large-Scale Pedigree Analysis — Supplementary Data 

# Reducing Pervasive False-Positive Identical-by-Descent Segments Detected by Large-Scale Pedigree Analysis

## Supplementary Data

files

**Files in this Data Supplement:**

- Supplementary Data - pdf file
- Supplementary Data - txt file
