# Supplementary material for: Reducing Pervasive False-Positive Identical-by-Descent Segments Detected by Large-Scale Pedigree Analysis
Source: Mol Biol Evol. 2014 Apr 30;31(8):2212–22. doi: 10.1093/molbev/msu151 (PMC4104314; doi:10.1093/molbev/msu151)
Supplement: Supplementary Data [file supp_msu151_suppl.pdf]

Supplementary Material for Reducing pervasive false positive identical-by-descent segments detected by large-scale pedigree analysis

Eric Y. Durand[1,†], Nicholas Eriksson[1], Cory Y. McLean[1,†]
1 23andMe, Inc., Mountain View, CA, USA
† These authors contributed equally to this work.
Corresponding author: Cory Y. McLean, cmclean@23andme.com

Supplementary Note

Logarithm of Odds (LOD) segment scoring

In this section, we describe an alternative scoring for potential IBD segments that is similar in spirit to the LOD score used in RefinedIBD (Browning BL and Browning SR 2013). Specifically, for a given segment S shared between two individuals i_1 and i_2, we compute its LODscore as follows:

$$\text{LODscore}(S) = log\left(\frac{Pr\left(G^{(S)}_{obs1}, G^{(S)}_{obs2}|\text{IBD}\right)}{Pr\left(G^{(S)}_{obs1}, G^{(S)}_{obs2}|\text{no IBD}\right)}\right), \quad (1)$$

where $G^{(S)}_{obs1}$ (resp. $G^{(s)}_{obs2}$) is the observed genotype of individual i_1 (resp. i_2) over segment S, and $Pr\left(G^{(S)}_{obs1}, G^{(S)}_{obs2}|\text{IBD}\right)$ (resp. $Pr\left(G^{(S)}_{obs1}, G^{(S)}_{obs2}|\text{no IBD}\right)$) is the pseudo-likelihood of observing $G^{(S)}_{obs1}$ and $G^{(S)}_{obs2}$ conditioned on individuals i_1 and i_2 being IBD over segment S (resp. not being IBD).

The pseudo-likelihood is computed as follows:

$$Pr\left(G^{(s)}_{obs1}, G^{(s)}_{obs2}|\text{IBD}, \epsilon\right) =$$
$$\prod_{i=1}^{\#S} \sum_{G_{true1}} \sum_{G_{true2}} Pr\left(G^{(i)}_{true1}, G^{(i)}_{true2}|\text{IBD}\right) Pr\left(G^{(i)}_{true1}|G^{(i)}_{obs1}, \epsilon\right) Pr\left(G^{(i)}_{true2}|G^{(i)}_{obs2}, \epsilon\right),$$

where ϵ is the genotyping error rate, $\#S$ is the number of markers in the IBD segment, and $G^{(i)}_{true\text{j}}$ is the true genotype of individual j at position i. The probability of genotypes $(G^{(i)}_{true1}, G^{(i)}_{true2})$ as a function of the IBD state (0, 1 or 2 alleles shared IBD at position i) is given in **Supplementary Table S3**. We note that **Supplementary Table S3** was derived elsewhere (Albrechtsen et al. 2009). The probability of observing a genotype given the true genotype and the genotyping error rate is given in **Supplementary Table S4**. Two genotypes are considered IBD if they either share one or two alleles IBD (IBD1 and IBD2 in **Supplementary Table S4**), and we give equal prior probabilities to the two configurations.

We assessed the performance of LODscore by computing its AUC for various segment sizes. We note that even though the LODscore has power to filter out false IBD segments, its AUC is generally lower than the HaploScore detailed in the main text (**Supplementary Figure S10**). Reasons for the lower power of LODscore may arise in part from two issues: 1) LODscore assumes each site is independent and thus ignores correlation between adjacent markers, and 2) LODscore ignores available phase information. Both issues could be alleviated by explicitly incorporating linkage disequilibrium between adjacent sites and switch errors into the model. However, because of the strong performance of HaploScore, we did not explore these research avenues further.

Supplementary References

Albrechtsen A, Sand Korneliussen T, Moltke I, van Overseem Hansen T, Nielsen FC, Nielsen R. 2009. Relatedness mapping and tracts of relatedness for genome-wide data in the presence of linkage disequilibrium. Genetic Epidemiology. 33:266–274.

Browning BL, Browning SR. 2013. Improving the accuracy and efficiency of identity-by-descent detection in population data. Genetics. 194:459–471.

Supplementary Figures

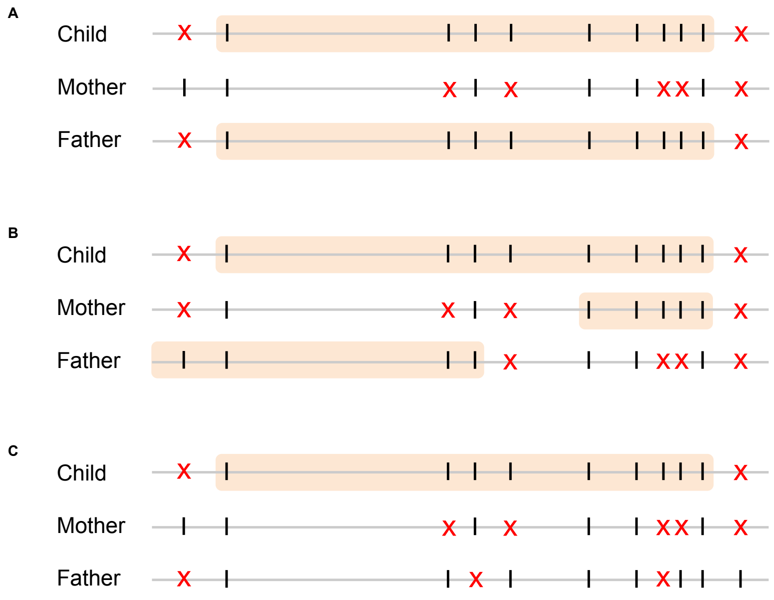

Figure S1. Choosing the parent through which child-other IBD segments have been transmitted. The genome is represented as a horizontal gray line. Assayed sites compatible with IBD between the listed individual and a hypothetical other individual (not pictured) are indicated as vertical black lines. Assayed sites incompatible with IBD (e.g., opposite homozygote sites) are indicated as red crosses. Orange boxes indicate reported IBD segments between the listed individual and the hypothetical other individual (not pictured). **A.** The unambiguous case in which one parent has a corresponding IBD segment and the other parent does not. Here, the father would be selected as the parent for analysis. **B.** The case where each parent has an IBD segment that partially overlaps the child segment. The parent selected for analysis is determined by the fraction of sites shared IBD. In this case, despite the longer physical length of the father's segment, the mother would be selected since her segment overlap (5 of 9 sites) is larger than the father's (3 of 9 sites). **C.** The case where neither parent has a reported IBD segment. The father would be selected as the parent for analysis, since his genotype contains fewer opposite homozygote sites in the child IBD region.

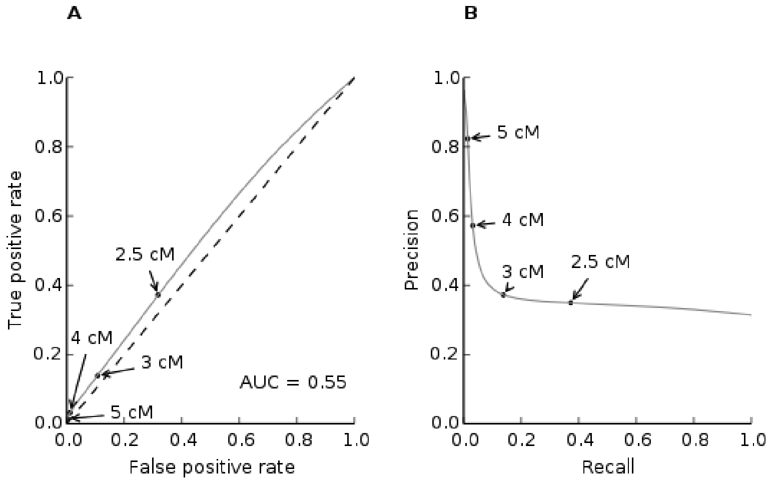

Figure S2. Receiver operating characteristics of child-other IBD segments discriminating by genetic length. A. True positive rate vs. false positive rate when discriminating by minimum genetic length. **B.** Precision vs. recall when discriminating by minimum genetic length. Values for four particular minimum genetic length criteria are marked on each plot.

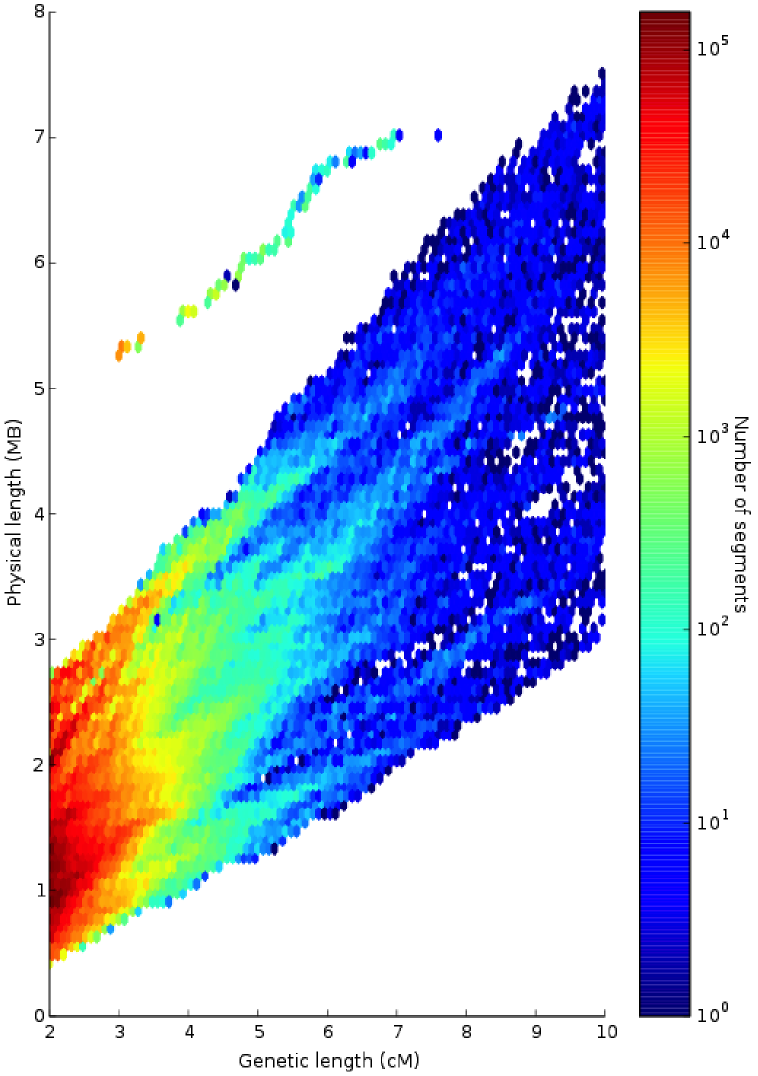

Figure S3. Length distribution of child-other IBD segments. Heat map shows the number of segments in each bin segregating by the genetic and physical lengths of the segments. Axes identical to those in **Figure 2A**.

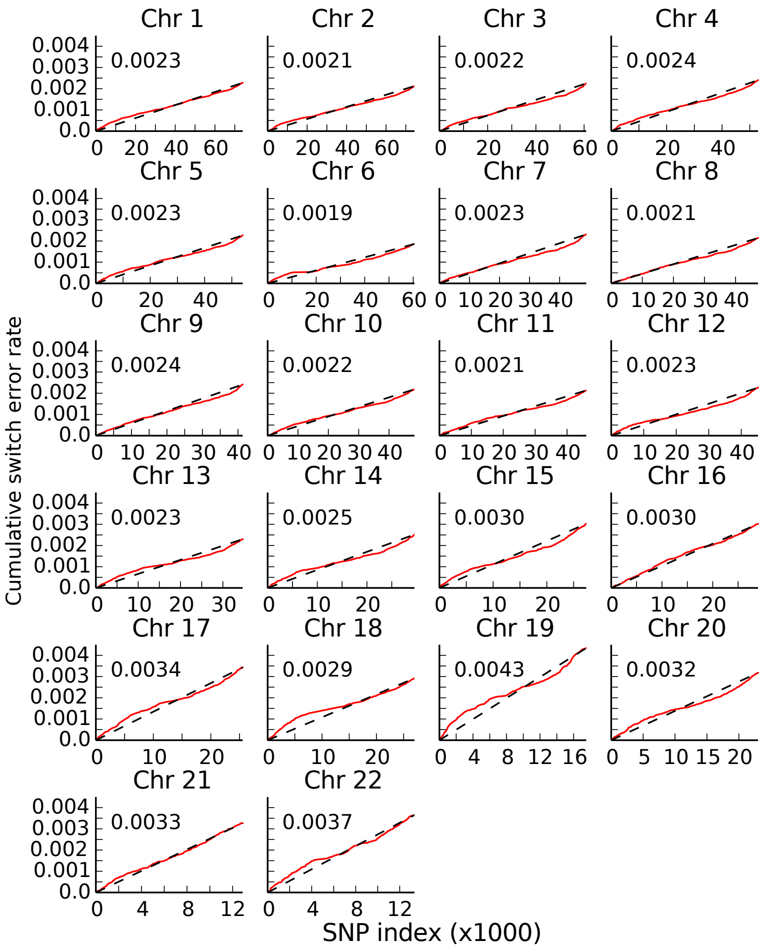

Figure S4. Switch errors in BEAGLE-phased data occur at a nearly-constant rate across chromosomes. Switch error positions were detected in 2,952 trio children by comparing BEAGLE-phased haplotypes with trio-phased haplotypes and assuming trio-phased data was truth. The average individual switch error rate was calculated at each site by dividing the total number of switch errors at that site by 2,952. Red lines plot the cumulative switch error rate scaled by the number of sites on the chromosome, to facilitate inter-chromosomal comparison. Numbers in the top left of each graph indicate the average per-site switch error rate for the chromosome. Black dashed lines indicate the expected individual cumulative switch errors per site assuming a constant switch error rate at each site on the chromosome.

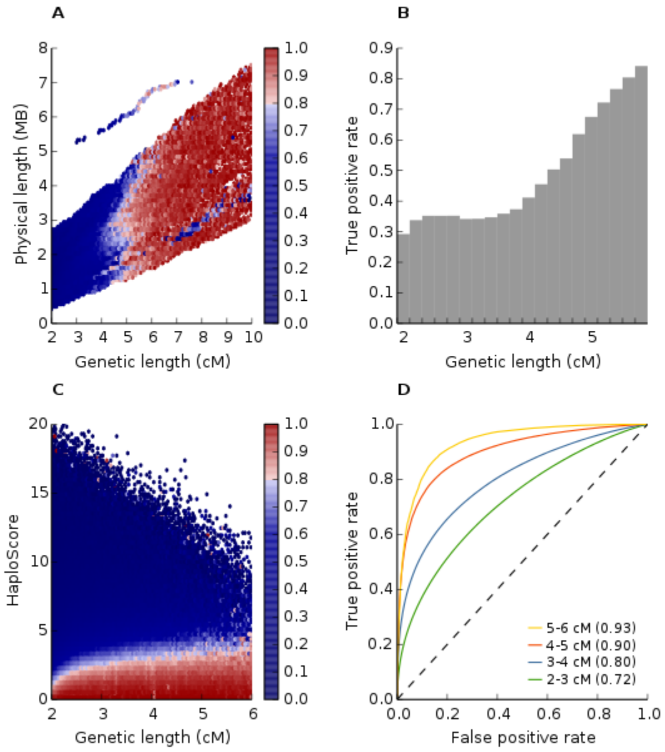

Figure S5. IBD segment overlap and HaploScore performance on chromosome 21 using trio-phased trios. A. Heat map of the mean fraction of reported IBD segments found in parents, binned by two measures of segment length. **B.** The fraction of child-other segments that are true IBD as a function of segment length. True IBD segments are defined as having at least 80% of their sites encompassed by a parent-other segment. **C.** Heat map of the mean fraction of reported IBD segments found in parents, binned by segment genetic length and HaploScore. **D.** Receiver operating characteristic for reported IBD segments of various lengths, discriminating by HaploScore. The four panels are analogous to **Figure 2A**,**B** and **Figure 4A**,**B**, respectively, using trio-phased data for all 2,952 trios. The similarity of this figure and the main text figure panels indicates that haplotype phasing errors do not contribute substantially to the estimates of IBD accuracy.

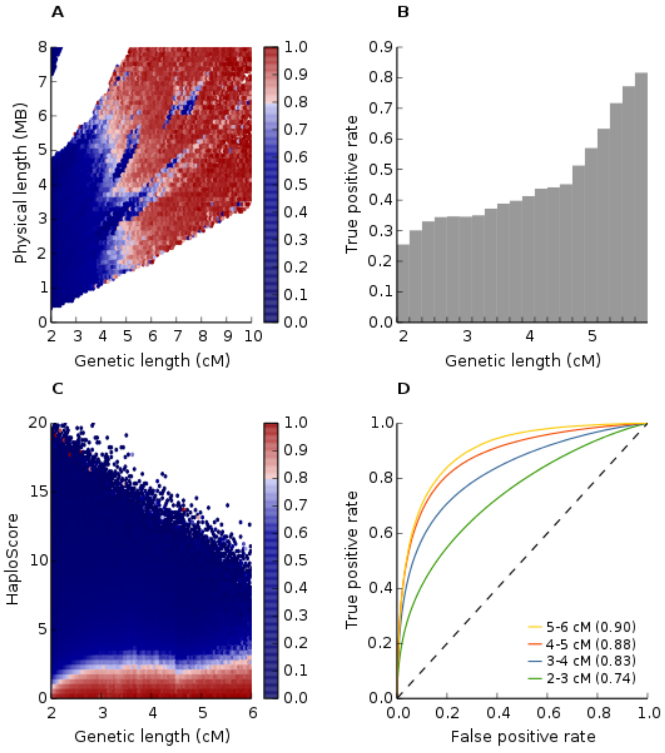

Figure S6. IBD segment overlap and HaploScore performance on chromosome 10. A. Heat map of the mean fraction of reported IBD segments found in parents, binned by two measures of segment length. **B.** The fraction of child-other segments that are true IBD as a function of segment length. True IBD segments are defined as having at least 80% of their sites encompassed by a parent-other segment. **C.** Heat map of the mean fraction of reported IBD segments found in parents, binned by segment genetic length and HaploScore. **D.** Receiver operating characteristic for reported IBD segments of various lengths, discriminating by HaploScore. The four panels are analogous to **Figure 2A**,**B** and **Figure 4A**,**B**, respectively, calculated on chromosome 10 here.

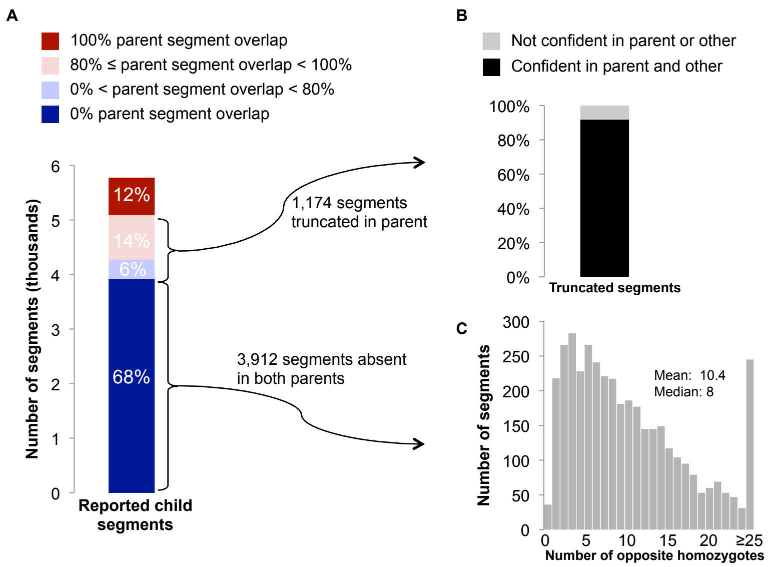

Figure S7. Analysis of child-other segments in parents in the 1000 Genomes cohort. This figure is analogous to **Figure 1** but performed on the 1000 Genomes cohort. **A.** The majority of child-other segments are not detected in either parent. **B.** Truncation points for parent-other segments are nearly always confidently-genotyped opposite homozygote sites, consistent with false positive IBD in the child. The opposite homozygote site causing truncation of the parent-other segment was examined in all 1,174 segments with partial parent overlap. **C.** Child-other segments with no corresponding parent-other segments contain many parent-other opposite homozygotes in the region, also consistent with false positive IBD in the child. For each of these child-other segments, the number of opposite homozygote sites present between the parent and the other individual at that segment location is calculated separately for each parent, and the smaller is chosen as the number of opposite homozygotes in the region.

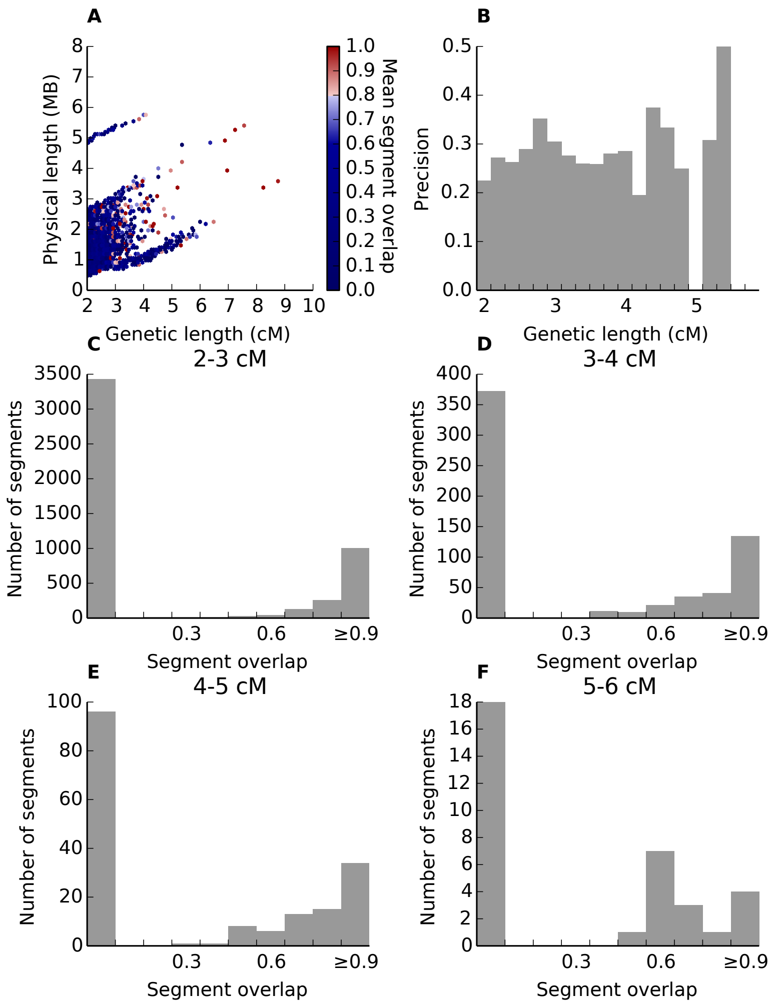

Figure S8. Accuracy of child-other IBD segments reported by GERMLINE in the 1000 Genomes cohort. This figure is analogous to **Figure 2** but performed on the 1000 Genomes cohort. **A.** Heat map of the mean fraction of reported child-other IBD segments contained in a corresponding parent-other segment, binned by two measures of segment length as described in **Figure 2A**. **B.** The fraction of child-other segments that are true IBD as a function of segment length. True IBD segments are defined as having at least 80% of their sites encompassed by a parent-other segment as in **Figure 2B**. **C–F.** Histograms of child-other segment counts binned by segment overlap for segments of 2–3 cM (**C**), 3–4 cM (**D**), 4–5 cM (**E**), and 5–6 cM (**F**). Note the scale changes on the y-axes: though the fraction of true segments of length < 3 cM is smallest, this range contains over 5-fold more true segments than all other length ranges combined.

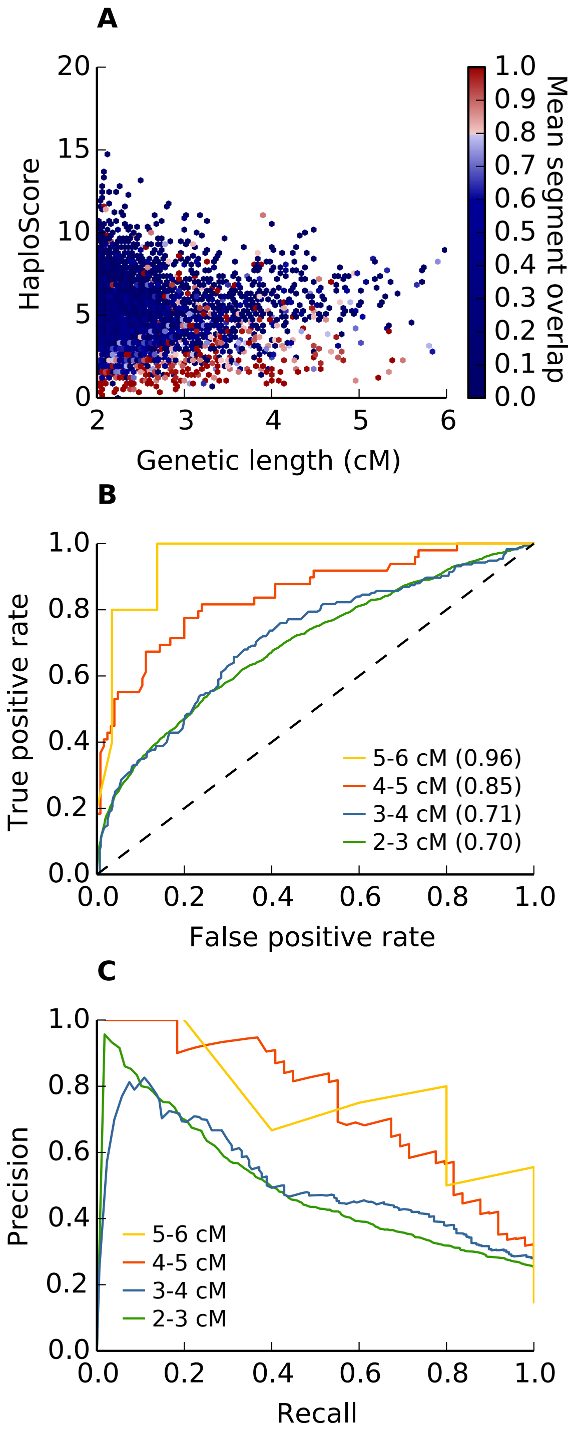

Figure S9. Improving detection of true IBD segments using HaploScore in the 1000 Genomes cohort. This figure is analogous to **Figure 4** but performed on the 1000 Genomes cohort. **A.** Heat map of the mean fraction of reported IBD segments found in parents, binned by segment genetic length and HaploScore. Calculations are performed as in **Figure 2A**. **B.** Receiver operating characteristic for reported IBD segments of various lengths, discriminating by HaploScore. True IBD is defined as in **Figure 2B**. The dashed black line indicates the no-discrimination line. The area under each curve is parenthesized in its legend entry. **C.** Precision-recall plot for child-other segments binned by segment length.

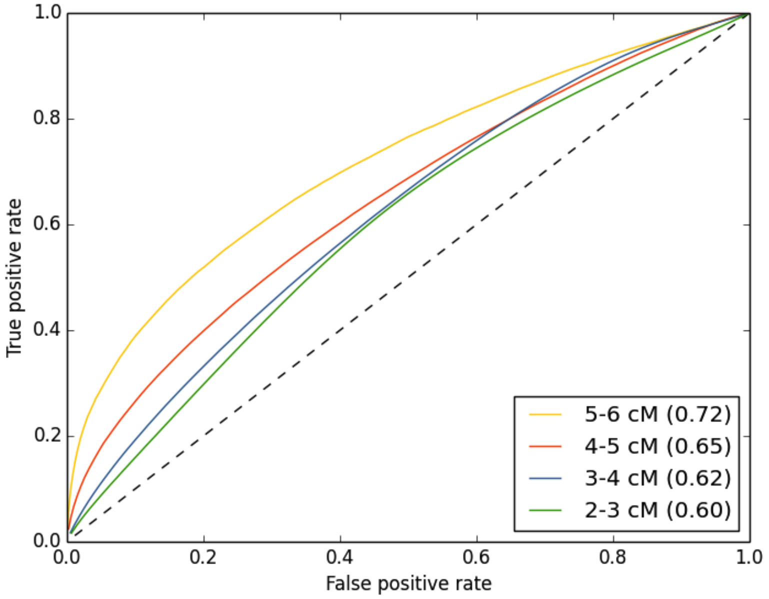

Figure S10. Receiver operating characteristic for reported IBD segments of various lengths, discriminating by LODscore. True positive IBD segments are defined as having at least 80% of their sites encompassed by a parent-other segment. The area under each curve is parenthesized in its legend entry.

Supplementary Tables

Table S1. Characteristics of the individuals in the 1000 Genomes cohort.

Population[a]	Total trios	Total individuals	Reported relationships
IBS	50	150	All trios
CEU	2	104	Two trios, 98 unrelated[b]
GBR	0	101	One mother-daughter pair, one unknown second order relationship, 97 unrelated
TSI	0	100	One sibling pair, 98 unrelated
FIN	0	100	All unrelated

[a] IBS, Iberian populations from Spain; CEU, Utah residents with ancestry from northern and western Europe; GBR, British from England and Scotland; TSI, Toscans from Italy; FIN, Finnish from Finland.
[b] Reportedly unrelated NA06989 and NA12155 share 35 cM on chromosome 21.

Table S2. Haplotype and diplotype window matches in child-other segments of 1000 Genomes data. A. Counts of window types in windows contained within a corresponding parent-other segment. **B.** Counts of window types in windows that are not contained within a corresponding parent-other segment.

A

	Child Diplo	Child Haplo	Child Both	*Total*
**Par None**	0	0	0	*0*
**Par Diplo**	3,257	53	798	*4,108*
**Par Haplo**	50	167	170	*387*
**Par Both**	817	169	7,702	*8,688*
Total	*4,124*	*389*	*8,670*	*13,183*

B

	Child Diplo	Child Haplo	Child Both	*Total*
**Par None**	7,955	424	4,397	*12,776*
**Par Diplo**	6,037	102	1,914	*8,053*
**Par Haplo**	90	281	387	*758*
**Par Both**	1,267	278	8,227	*9,772*
Total	*15,349*	*1,085*	*14,925*	*31,359*

Par, parent; Diplo, diplotype match only; Haplo, haplotype match only.

Table S3. Genotype probabilities for a pair of individuals for different IBD states.

(G_1, G_2)	**IBD0**	**IBD1**	**IBD2**
AA, BB	$2p^2q^2$	0	0
AA, AA	p^4	p^3	p^2
AA, AB	$4p^3q$	$2p^2q$	0
AB, AB	$4p^2q^2$	$p^2q + pq^2$	$2pq$

p represents the allele frequency of allele A and q $(= 1 - p)$ represents the allele frequency of allele B.

Table S4. Observed genotype probabilities with genotyping errors.

	$G_{true} = AA$	$G_{true} = AB$	$G_{true} = BB$
$G_{obs} = AA$	$(1-\epsilon)^2$	$(1-\epsilon)\epsilon$	ϵ^2
$G_{obs} = AB$	$2(1-\epsilon)\epsilon$	$(1-\epsilon)^2 + \epsilon^2$	$2(1-\epsilon)\epsilon$
$G_{obs} = BB$	ϵ^2	$(1-\epsilon)\epsilon$	$(1-\epsilon)^2$
